# Beyond enteral feedings: Teduglutide and its role in gut resiliency

**DOI:** 10.1002/jpr3.70292

**Published:** 2026-11-10

**Authors:** Heather Grabow, Michael Duffy, David H. Seto, Paula Prieto Tizzard

**Affiliations:** ^1^ Department of Gastroenterology Phoenix Children's Phoenix Arizona USA

**Keywords:** enterocytes, GLP‐2 analog, intestinal immunity, short bowel syndrome

## Abstract

Teduglutide is a glucagon‐like peptide‐2 (GLP‐2) analog that promotes intestinal adaptation with a goal of reducing dependence on parenteral nutrition (PN) in those with short bowel syndrome (SBS). We describe a 2‐year‐old, born at 26 weeks gestation, with a history of PN dependency due to recurrent necrotizing enterocolitis (NEC) and secondary small bowel resections resulting in 90 cm of small bowel (without ileocecal valve, intact colon), who demonstrated a remarkable response to teduglutide. The patient herein had full discontinuation of PN in 1 month with enhanced immunity to gastrointestinal (GI) illness with the use of GLP‐2 analog. This case offers new insight into teduglutide, potentially augmenting intestinal immunity. This patient revealed heightened response to GI infections while on teduglutide, yet poor tolerance to the same infection with missed dosing. To our knowledge, this is the first report on teduglutide possibly augmenting immunity.

## 1 INTRODUCTION

Teduglutide is a glucagon‐like peptide‐2 (GLP‐2) analog that was approved in 2012 for the treatment of short bowel syndrome (SBS) in adults and in 2019 for its use in children 1 year of age and older. This medication promotes intestinal adaptation through villi growth to reduce parenteral nutrition (PN) dependence in those with SBS.^1^ Teduglutide works via GLP‐2 agonism, which results in crypt stem cell activation, promoting enterocyte replication, and preventing enterocyte apoptosis; therefore, increasing mucosal epithelium volume and surface area. GLP‐2 agonism also enhances the protein barrier function of enteric tight junctions and optimizes mesenteric blood flow, allowing for strong enteric integrity.^2^, ^3^ Immunologically, GLP‐2 activates anti‐inflammatory cytokines including interleukin‐4, interleukin‐10 and insulin‐like growth factor‐1; promoting T‐helper 2 (Th2) mediated anti‐inflammatory protection. Additionally, it suppresses the nuclear factor kappa‐light‐chain‐enhancer of activated B cells (NF‐κB) and blocks phosphorylation of extracellular signal‐regulated kinase, inhibiting pro‐inflammatory tumor necrosis factor‐alpha (TNF‐α), interleukin‐beta, and interleukin‐6 (IL‐6).^4^ Of note, these studies were in vitro studies on mice or human cells, as currently there is little research on the immune effects of GLP‐2 analogs in human studies.

Ultimately, the goals of teduglutide are to decrease the volume of PN or achieve enteral autonomy.^5^ Historically, those impacted by this disease are PN dependent and, without increasing the absorptive intestinal abilities, are at risk of death, central access complications, or debilitating treatment (e.g., small bowel transplant).^6^ Our patient displayed a strong response to teduglutide upon initiation, yet, with a single missed dose and ultimately five total missed doses, suffered rapid inability to tolerate enteral nutrition (EN) and a significant increase in stool output while also ill with rhino/enterovirus. In the setting of both illness and medication lapse, rapid symptom onset was evident yet unclear if due to missed treatment or lost protective GLP‐2 enterocyte modulation.

## 2 CASE REPORT

We present a 2‐year‐old born at 26 weeks, with a history of necrotizing enterocolitis (NEC) and subsequent enterotomies, with remaining 90 cm of small bowel (without an ileocecal valve) and intact colon, PN dependence, and feeding intolerance. Given multiple central line infections, our patient had no remaining patent vasculature for central access, prompting temporary placement of a left internal jugular vein catheter while progressing toward small bowel transplant. In the meantime, teduglutide 0.05 mg/kg subcutaneously once daily was initiated.

Our patient's maximum tolerated enteral feed prior to initial treatment with teduglutide and while 35% PN dependent was 1 kcal/mL amino acid formula + 10 mL/day medium‐chain triglyceride emulsion (MCT) at 31 mL/h (~65% of goal). She was able to be transitioned off parenteral support after 35 days of teduglutide, along with having the patient's central line removed.

After 2 months of teduglutide and multiple viral infections (e.g., rhino/enterovirus and rotavirus), she remained exclusively enterally fed on a 1 kcal/mL amino acid formula + 10 mL/day MCT given continuously at 48 mL/h. Baseline stool output averaged ~12–15 mL/kg/day.

Four months after starting teduglutide and 3 months after PN discontinuation, our patient presented with new‐onset emesis and non‐bloody diarrhea requiring hospital admission, testing positive again for rhino/enterovirus via nasopharyngeal (NP) swab polymerase chain reaction (PCR). During this time, the patient missed a total of 5 days of teduglutide due to an issue with medication access secondary to an insurance denial. With this second diarrheal illness, and while off teduglutide, she reached a maximum stool output of ~60 mL/kg/day, needing temporary discontinuation of EN, bowel rest, and 1.5× intravenous fluids due to significantly increased stool output, electrolyte derangements, and weight loss. She had resolution of GI symptoms and was back on the previously tolerated enteral feed rate 3 days after resuming teduglutide (Figure 1). No probiotics were used.

**Figure 1:**
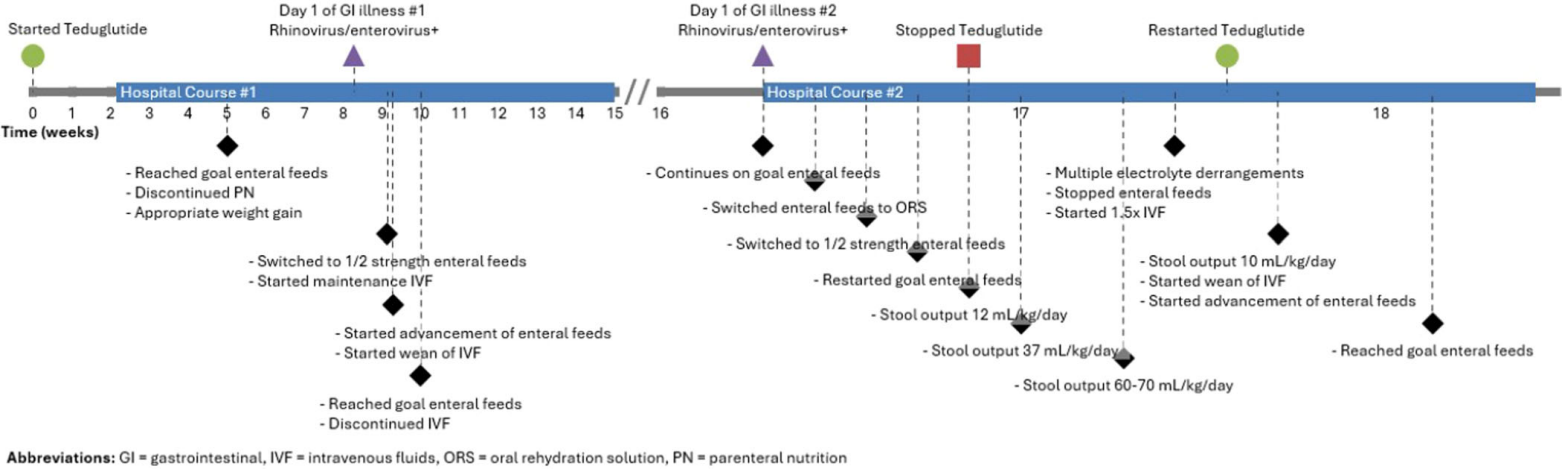
Timeline of events. GI, gastrointestinal; IVF, intravenous fluids; ORS, oral rehydration solution; PN, parenteral nutrition.

## 3 DISCUSSION

This case highlights an interesting discussion of exacerbated response to a viral infection with a brief lapse in teduglutide use. Median response time to teduglutide in adults is 4.3 months and is defined as ≥20% reduction in parenteral support.^7^ Data for the pediatric population are limited. Our patient had a rapid response at the age of 2 years. Pediatric patients have been shown to remain in remission with continued tolerance of EN while on teduglutide.^8^ There is limited literature discussing an expected timeline for return of symptoms after missed dosing. In the setting of a missed dose, the manufacturer advises against duplicate dosing, seemingly allowing 1 missed dose without presumable harm. The half‐life of teduglutide is 0.7 ± 0.2 h in children less than 12 years, and crypt stem cell activation for enterocyte proliferation is initiated within this short amount of time. This process is initiated within teduglutide's short half‐life; then proliferated enterocytes have a 3‐ to 5‐day life cycle. Therefore, theoretically, one missed dose should not significantly impact symptoms in a noninfectious intestine.

In the context of enterovirus infections that cause severe diarrhea, villus atrophy, and decreased GLP‐2 levels, GLP‐2 effects can combat the negative impacts by promoting epithelial regeneration, repairing damage, and reducing inflammation.

GLP‐2 promotes an increase in villus height and crypt depth, which can aid in reversing atrophy caused by viral infection. It also increases the expression of tight junction proteins (zonula occludens‐1, occludin, and claudin‐1), increasing permeability and decreasing bacterial translocation that occurs with enterovirus‐induced diarrhea. The presence of GLP‐2 also inhibits apoptosis in enterocytes, reduces expression of proinflammatory cytokines (TNF‐α and IL‐6), while elevating anti‐inflammatory cytokines and increasing intestinal blood flow via nitric oxide (NO) pathways, providing the necessary resources for tissue repair after infection.

## 4 CONCLUSION

The connection between teduglutide use and heightened intestinal immunity is feasible, with the knowledge of GLP‐2 agonism in enteric immunity, anti‐inflammatory responses, promoting anti‐apoptotic actions, blood flow, intestinal barrier function, and stimulating mucosal regeneration. Between the known mechanisms of GLP‐2 agonism in enteric immunity and this case report, there is a possibility that teduglutide may play a role not only in increasing bowel absorptive area but also enhancing enteric immunity. More studies are needed to further expand on the potential immune role of teduglutide in SBS patients.

## CONFLICT OF INTEREST STATEMENT

The authors declare no conflicts of interest.

## ETHICS STATEMENT

The authors thank the patient's parents for providing their consent to publish this work.
